# The transcription factor THAP11 is a general regulator of early embryonic polarization

**DOI:** 10.64898/2026.09.21.753235

**Published:** 2026-11-04

**Authors:** Qianying Yang, Shan Jiang, Boyan Wang, Yi Zhang

## Abstract

During mouse embryogenesis, three sequential waves of cell polarization occur in the 8-cell-morula trophectoderm (TE), E3.5-E4.5 primitive endoderm (PrE), and E4.5-E5.5 epiblast (Epi), driving cell fate determination and morphogenic events that lay the foundation for embryonic development. However, it is not clear whether a general regulator that functions in all three waves of polarization exists. Here, we demonstrate that transcription factor THAP11 is such a general regulator. Using a novel mouse model combining genetic knockout and an inducible degron system, we demonstrate that stage-specific acute THAP11 depletion causes the failure of the three waves of cell polarization, resulting in defective blastocyst formation, PrE maturation, and Epi lumenogenesis, respectively. Mechanistically, THAP11 does not directly regulate pluripotency genes but drives polarization through both stage-specific and conserved chromatin-binding that commonly activate polarity programs, including Golgi vesicle transport. THAP11 activates polarity genes by promoting chromatin accessibility at their promoters. THAP11, together with stage-specific regulators, sequentially paces the timing of cell polarization. Collectively, our study establishes THAP11 as a general regulator of embryonic morphogenesis.

## Full Text Availability

The license terms selected by the author(s) for this preprint version do not permit archiving in PMC. The full text is available from the preprint server.

