# Supplementary material for: The transcription factor THAP11 is a general regulator of early embryonic polarization
Source: bioRxiv. 2026 Sep 22:2026.09.21.753235. Preprint. [Version 1] doi: 10.64898/2026.09.21.753235 (PMC13622157; doi:10.64898/2026.09.21.753235)
Supplement: Supplementary file 1 [file media-1.pdf]

# Supplementary Materials for

## The transcription factor THAP11 is a general regulator of early embryonic polarization

**The PDF file includes:**

Supplementary information, Fig. S1 to 10;
Supplementary Table 1 to 16 (separated files)
References

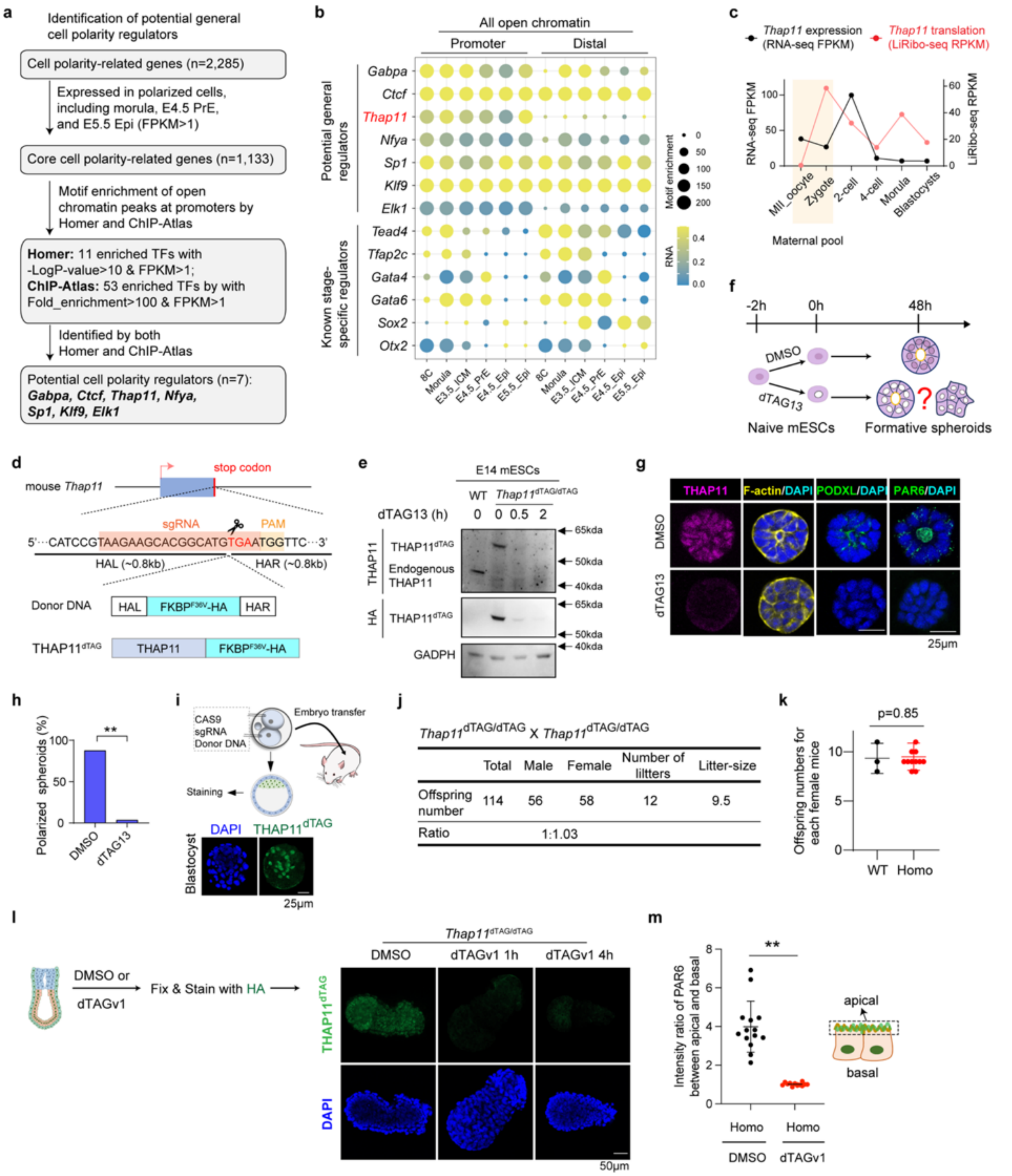


**Supplementary information, Fig. S1. Identifying THAP11 as a potential regulator of cell polarization and generation of THAP11-dTAG mouse line. a,** Schematic illustration for identifying candidate regulators underlying the three waves of cell polarization during early embryogenesis. **b,** Enrichment of TF motifs at promoter and distal ATAC–seq peaks of the

indicated stages. **c,** The dynamics of *Thap11* expression at transcription (RNA-seq, FPKM) and translation (LiRibo-seq [1], RPKM) in mouse oocytes and early embryos. **d,** Diagram illustration of HA-FKBP (F36V) knock-in at the 3' end of *Thap11* gene and the THAP11$^{dTAG}$ fusion protein. **e,** Western blot analysis confirming THAP11$^{dTAG}$ knock-in and dTAG13-mediated THAP11$^{dTAG}$ degradation in mES cells. The blots were incubated with anti-THAP11 and anti-HA, respectively. GAPDH was used as a loading control. **f,** Schematic illustration of the experimental design. **g,** Immunostaining of 48h 3D cultured mESCs with DMSO or dTAG13 treatment. Scale bar, 25 μm. **h,** The ratio of polarized spheroids. Chi-squared test, **p<0.01. **i,** Diagram illustration for *Thap11*$^{dTAG/dTAG}$ mouse generation and HA staining confirming the dTAG knock-in cells in blastocysts. Scale bar, 25 μm. **j,** Statistics of pup numbers from THAP11$^{dTAG/dTAG}$ × THAP11$^{dTAG/dTAG}$ crosses, indicating normal function of the THAP11$^{dTAG}$ fusion protein. **k,** Comparisons of pup numbers. Mean ± SEM; p-value, Student's t test. **l,** Left panel: experimental design. Right panel: Immunostaining of Homo embryos with or without dTAG13 treatment. **m,** Intensity ratio of PAR6 between apical and basal part in E5.5 Homo Epi with (n=12) or without (n=14) dTAGv1 treatment. ∗∗p < 0.01, Student's t test.

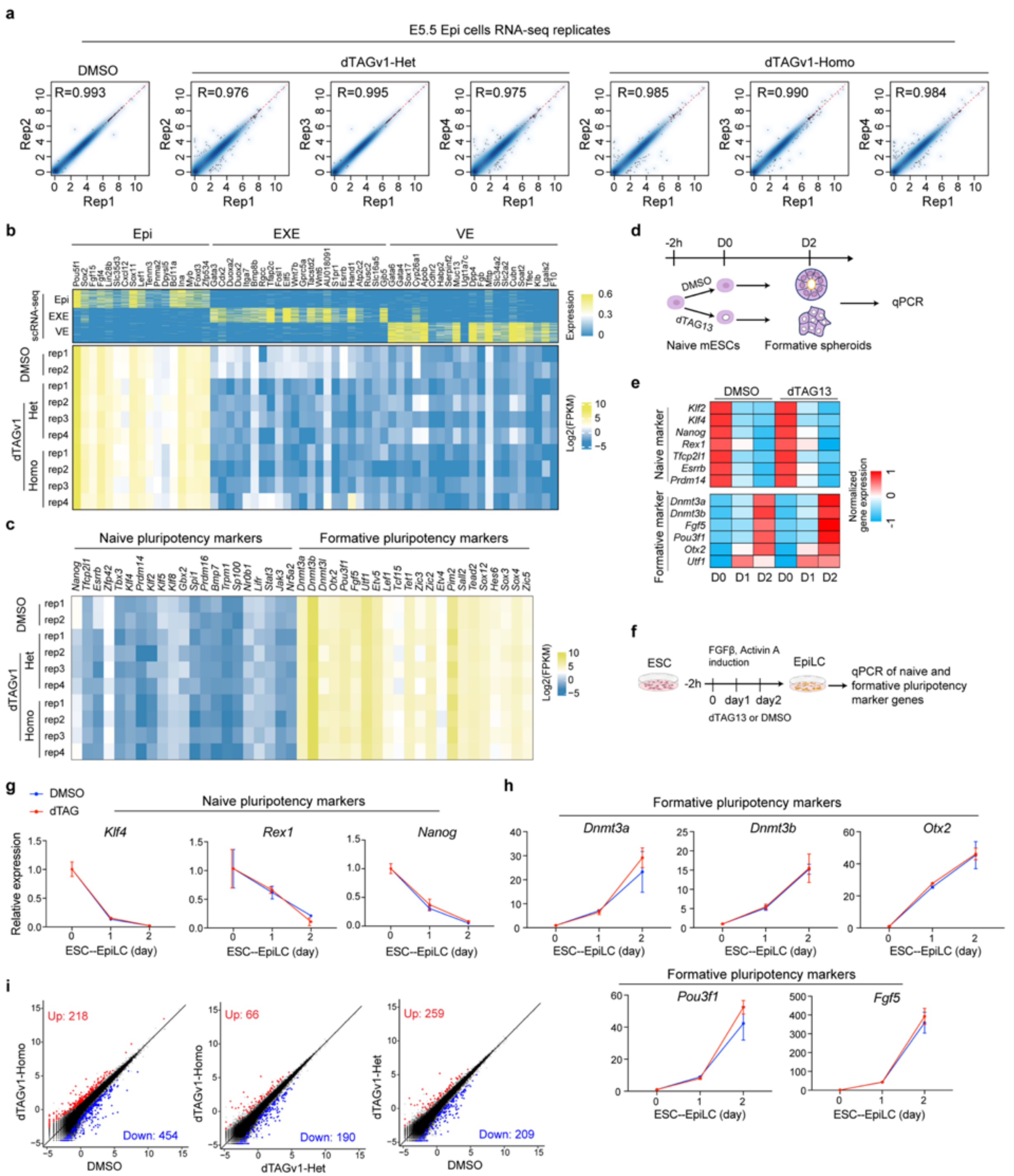


**Supplementary information, Fig. S2. THAP11 does not regulate pluripotency transition in epiblast cells. a,** Correlation of replicates for total RNA-seq in DMSO-*Thap11*$^{\mathrm{dTAG/dTAG}}$ (DMSO-Homo), dTAGv1-*Thap11*$^{\mathrm{dTAG/wt}}$ (dTAGv1-Het), and dTAGv1-*Thap11*$^{\mathrm{dTAG/dTAG}}$ (dTAGv1-Homo) E5.5 epiblast cells. The x and y axis of the dot plots are Log (CPM+1). **b,** Heatmap showing the expression of Epi-, ExE-, and VE-marker genes across single-cell RNA-seq datasets of Epi, ExE,

and VE [2], together with our bulk low-input total RNA-seq data from Epi samples. **c,** Heatmap showing the expression of naïve- and formative-pluripotency-marker genes based on bulk low-input total RNA-seq data of Epi samples. **d,** Schematic diagram of experimental design. **e,** Heatmap showing the expression dynamics of naïve- and formative-pluripotency-marker genes in 3D-cultured THAP11-dTAG ESCs with DMSO or dTAG13 treatment during induction. **f,** Schematic diagram of experimental design. **g-h,** Expression dynamics of naïve- and formative-pluripotency-marker genes in 2D-cultured THAP11-dTAG ESCs with DMSO or dTAG13 treatment during EpiLC differentiation. **i,** Scatter plots comparing gene expression profiles of E5.5 Epi cells from DMSO-Homo, dTAGv1-Het, and dTAGv1-Homo embryos. The x and y axis of the dot plots are Log2CPM (counts per million) from RNA-seq. Fold change > 2, false discovery rate (FDR) < 0.05.

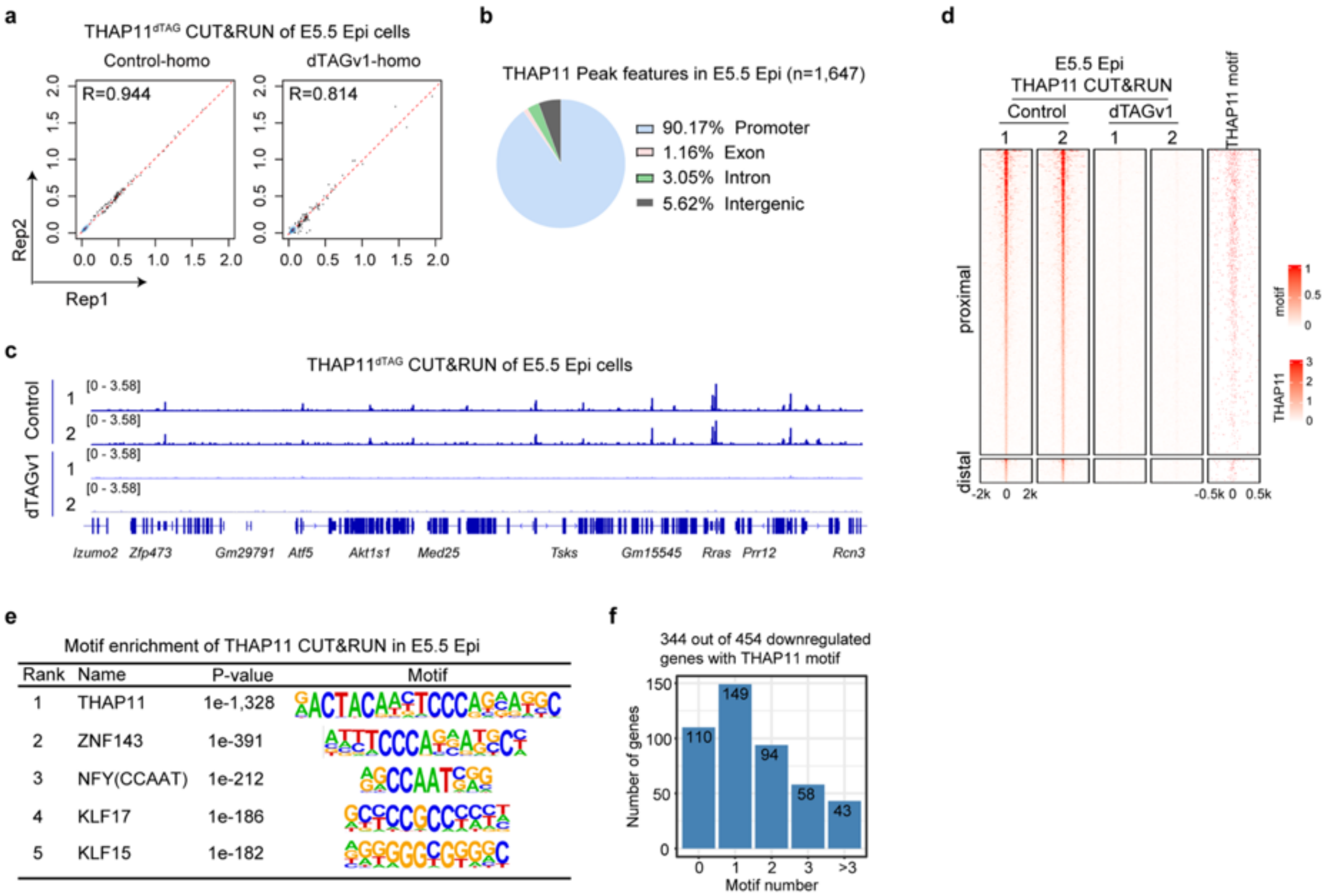


**Supplementary information, Fig. S3. THAP11 regulates polarity genes through promoter binding in epiblast cells. a,** Correlation of replicates for THAP11 CUT&RUN in E5.5 Epi cells. Each dot represents a 5-kb bin. **b,** The genomic distribution of THAP11 binding peaks generated by THAP11 CUT&RUN in E5.5 Epi cells. **c,** Genome browser view showing examples of THAP11 CUT&RUN profiles in E5.5 Epi cells with or without dTAGv1 treatment. **d,** Heatmaps showing all the CUT&RUN signals of THAP11 peaks at promoters and distal regions in E5.5 Epi cells with or without dTAGv1 treatment. **e,** The top 5 TF motifs enriched in the regions bound by THAP11 in E5.5 Epi cells. Note that based on the p-value, the THAP11 motif is much more highly enriched compared to the other enriched motifs. **f,** THAP11 motif numbers found in the promoters of the 454 downregulated genes (dTAGv1-Homo VS DMSO).

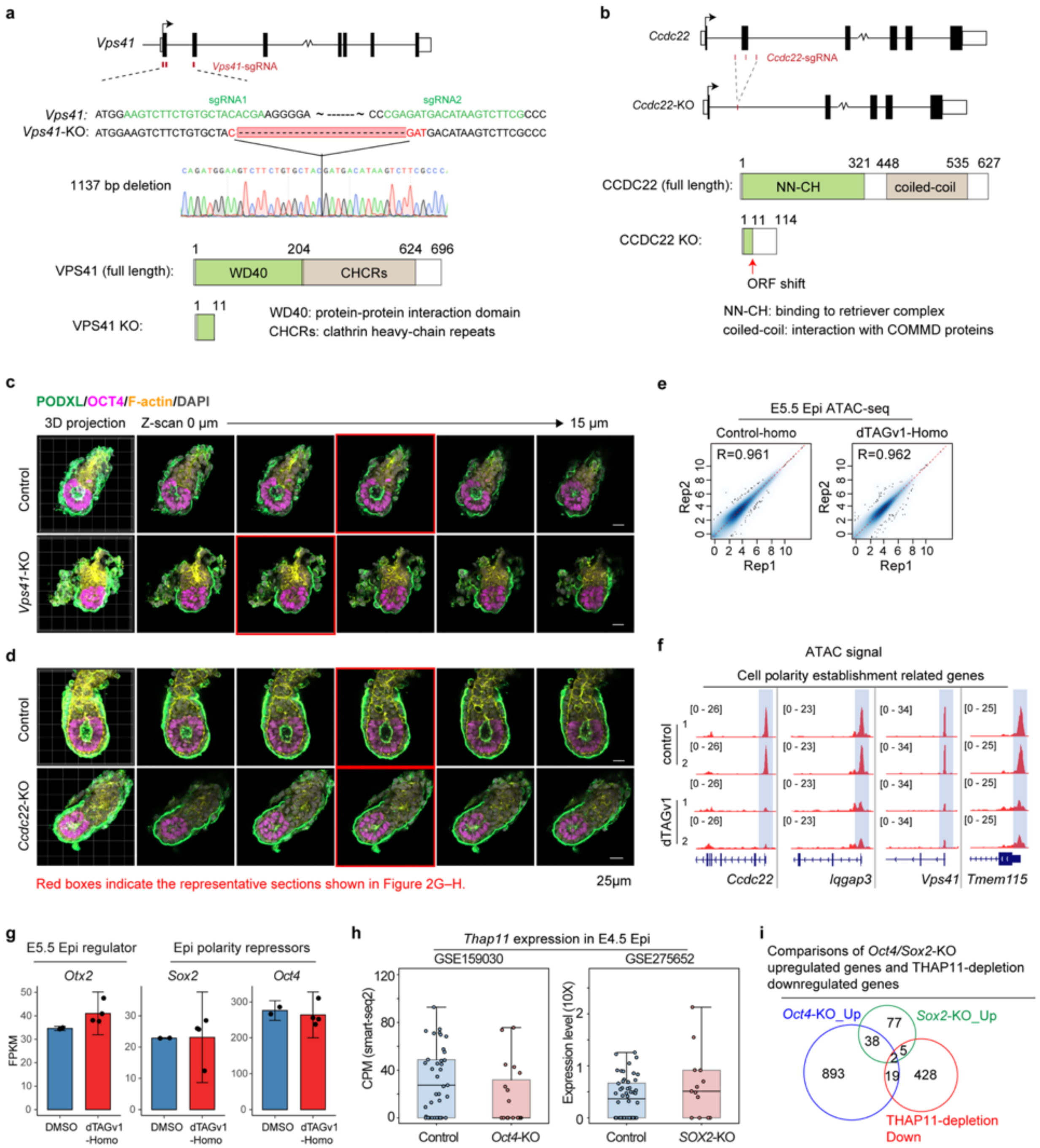


**Supplementary information, Fig. S4. *Vps41* and *Ccdc22* knockout impairs Epi lumenogenesis.** **a,** Schematic diagrams illustrating the *Vps41* knockout strategy, in which CRISPR-mediated deletion of exons 1–2, encoding the WD40 domain, results in premature translation termination. **b,** Schematic diagrams illustrating the *Ccdc22* knockout strategy, in which CRISPR-mediated deletion of exon 2, encoding the NH-CH domain, results in premature translation termination. **c-d,** Immunostaining of OCT4, F-actin, and PODXL in control, *Vps41*-KO (c), and *Ccdc22*-KO (d)

E5.5 embryos. The red boxes indicate the representative sections shown in Fig. 2g-h. Scale bar, 25 µm. **e,** Correlation of replicates for ATAC-seq in control or THAP11-depleted E5.5 Epi. The x and y axis of the dot plots are log2(normalized_counts+1). Each dot represents a 5-kb bin. **f,** Examples of genome browser view of ATAC signals in control and THAP11-depleted E5.5 Epi. **g,** Expression levels of *Otx2*, *Sox2*, and *Oct4* in control or THAP11-depleted E5.5 Epi cells. **h,** Expression levels of *Thap11* in E4.5 *Oct4*-KO (GSE159030) or *Sox2*-KO (GSE275652) Epi. **i,** Venn diagram showing the overlapped genes downregulated in the E5.5 THAP11-depleted Epi cells and upregulated in the E4.5 *Oct4*- or *Sox2*-KO Epi cells.

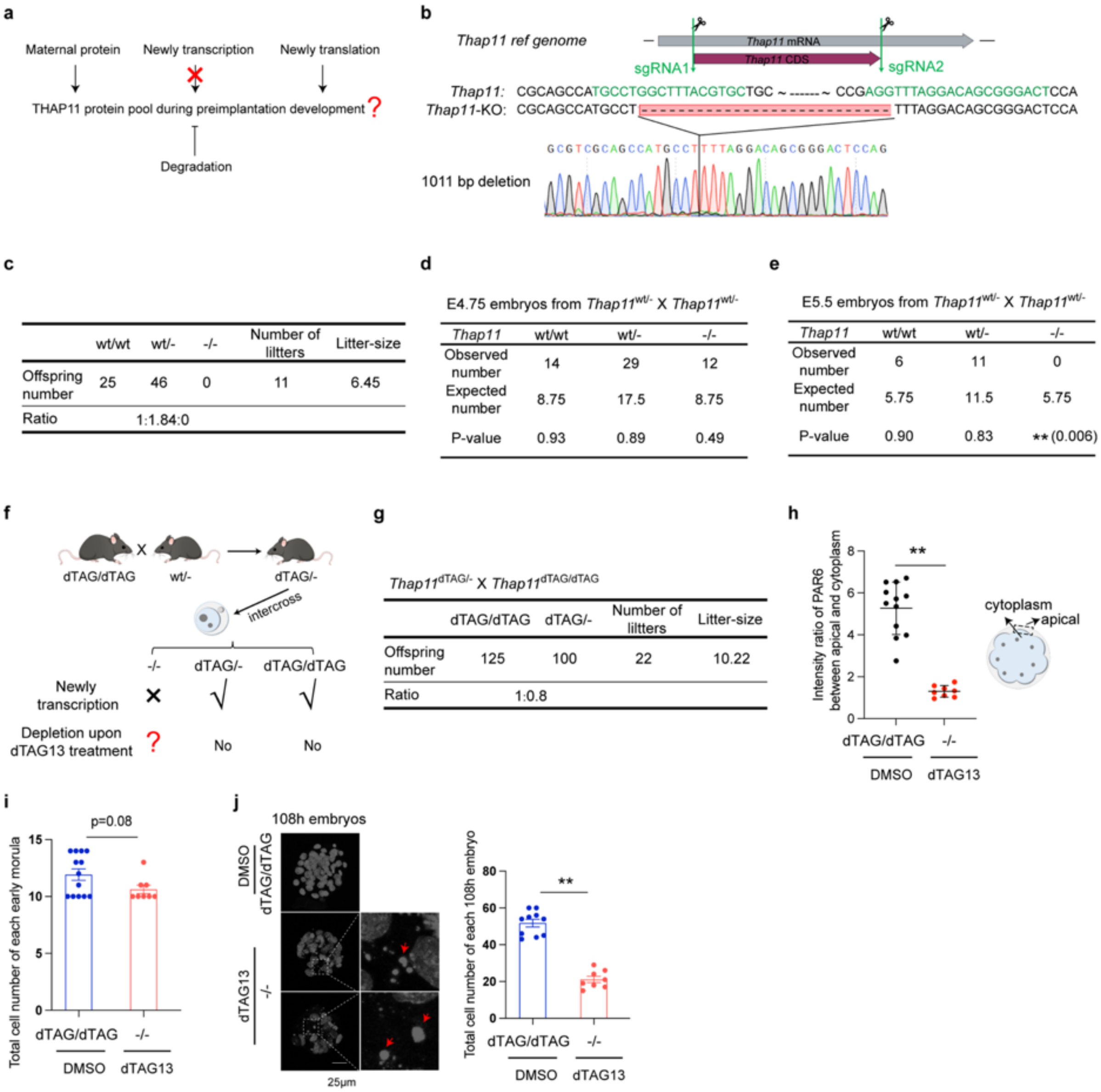


**Supplementary information, Fig. S5. Generation of the THAP11$^{dTAG/-}$ mouse line. a,** Schematic diagrams illustrating the THAP11 protein depletion strategy. **b,** Schematic diagrams illustrating the *Thap11* knockout strategy, in which CRISPR-mediated deletion of entire protein coding region. **c,** Statistics of pup numbers from THAP11$^{wt/-}$ × THAP11$^{wt/-}$ crosses, indicating successful generation of THAP11 knockout allele. **d,** Genotype ratios of E4.75 embryos from Thap11$^{+/-}$ intercrosses. Chi-square test. **e,** Genotype ratios of E5.5 embryos from Thap11$^{+/-}$ intercrosses. Chi-square test. **f,** The experimental design for the generation of THAP11-depleted embryos. **g,** Statistics of pup numbers from THAP11$^{dTAG/-}$ × THAP11$^{dTAG/dTAG}$ crosses, indicating normal function of the THAP11$^{dTAG}$ fusion protein. **h,** Intensity ratio of PAR6 between apical and

cytoplasm in control (n=12) or THAP11-depleted (n=8) early morula. $**p < 0.01$, Student's t test. **i,** Total cell number of control (n=13) and THAP11-depleted (n=8) early morulae. **j,** left panel: representative image of control and THAP11-depleted embryos at 108h. Scale bar, 25 μm. Red arrowheads indicate DNA fragmentation. Right panel: Total cell number of control (n=10) and THAP11-depleted (n=8) embryos at 108h. $**p < 0.01$, Student's t test.

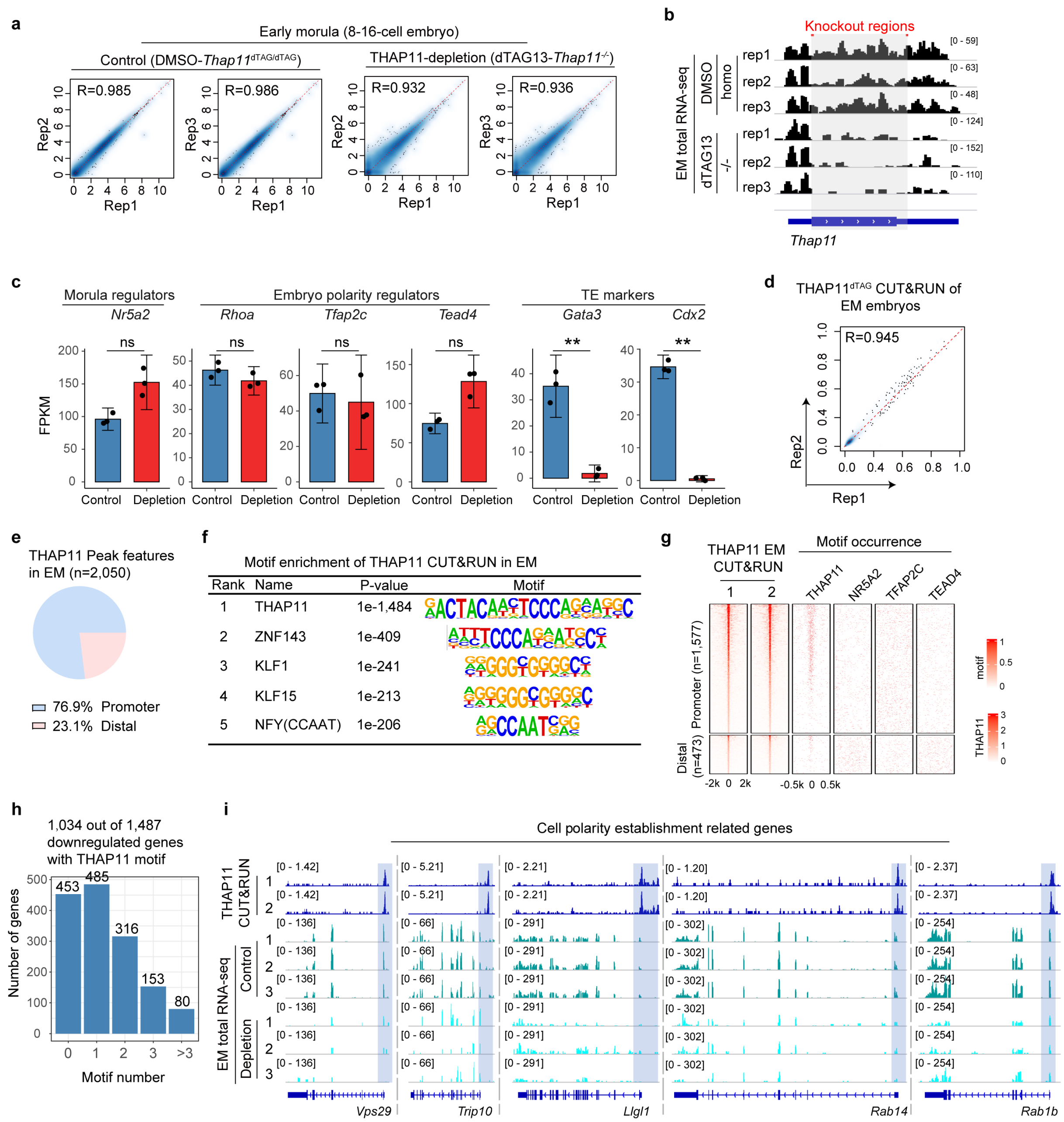


**Supplementary information, Fig. S6. THAP11 regulates polarity genes through promoter binding in EM. a,** Correlation of replicates for total RNA-seq in EM of control (DMSO-*Thap11*$^{\text{dTAG/dTAG}}$) and THAP11-depletion (dTAG13-*Thap11*$^{-/-}$). The x and y axis of the dot plots are Log (CPM+1). **b,** Genome browser view of RNA-seq at the *Thap11* genomic regions in EM of control and THAP11-depletion. **c,** Expression levels of *Nr5a2*, *Rhoa*, *Tfap2c*, *Tead4*, *Gata3*, and *Cdx2* in control or THAP11-depleted EM. **, FDR<0.01; ns, FDR>0.05. **d,** Correlation of replicates for THAP11 CUT&RUN in EM. Each dot represents a 5-kb bin. **e,** The genomic

distribution of THAP11 binding peaks generated by THAP11 CUT&RUN in EM. **f,** The top 5 TF motifs enriched in the regions bound by THAP11 in EM. Note that based on the p-value, the THAP11 motif is much more highly enriched compared to the other enriched motifs. **g,** Heatmaps showing all the CUT&RUN signals and motif occurrence of the THAP11 peaks at promoters and distal regions in EM. **h,** THAP11 motif numbers found in the promoters of the 1,487 downregulated genes without detectable CUT&RUN signal upon THAP11-depletion. **i,** Genome browser view of THAP11 targeted polarity gene examples showing THAP11 CUT&RUN and RNA-seq in EM.

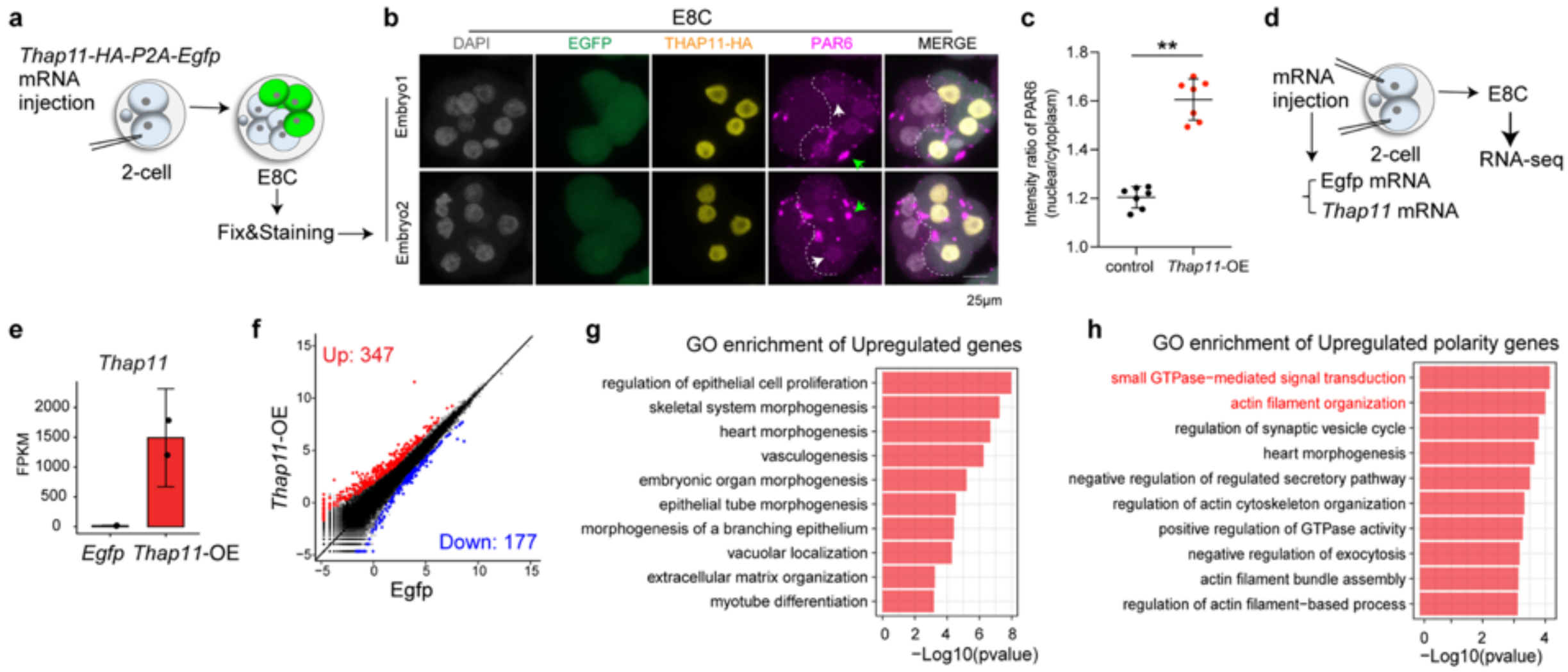


**Supplementary information, Fig. S7. THAP11 overexpression promotes premature clustering of apical proteins and activation of important polarity program. a,** Schematic diagram of experimental design. One blastomere of late 2-cell stage embryos was injected with Thap11-HA-P2A-Egfp mRNA, and the embryos were subsequently collected at early 8-cell (E8C) stage for immunostaining. **b,** Immunostaining of HA (THAP11-HA) and PAR6 in E8C embryos. White arrows indicate nuclear PAR6**.** Green arrows indicate membrane large PAR6 cluster. **c,** Intensity ratio of PAR6 between nuclear and cytoplasm in control or THAP11-OE blastomeres. Data are presented as mean ± SEM; $**p < 0.01$, Student's t test. **d,** Schematic diagram of experimental design. Late 2-cell stage embryos were injected with either Egfp- or Thap11-HA-P2A-Egfp mRNA, and embryos were subsequently collected at E8C stage for RNA-seq. **e,** Expression of Thap11 in Egfp- or Thap11-OE E8C embryos. **f,** Scatter plots comparing the gene expression profiles of E8C embryos with *Egfp* or *Thap11* overexpression. The x and y axis of the dot plots are $Log_2CPM$ from RNA-seq. Fold change > 2, FDR < 0.05. **g,** Representative GO terms enriched in the upregulated genes (n=347) in response to *Thap11* overexpression. **h,** Representative GO terms enriched in the upregulated polarity genes in response to *Thap11* overexpression.

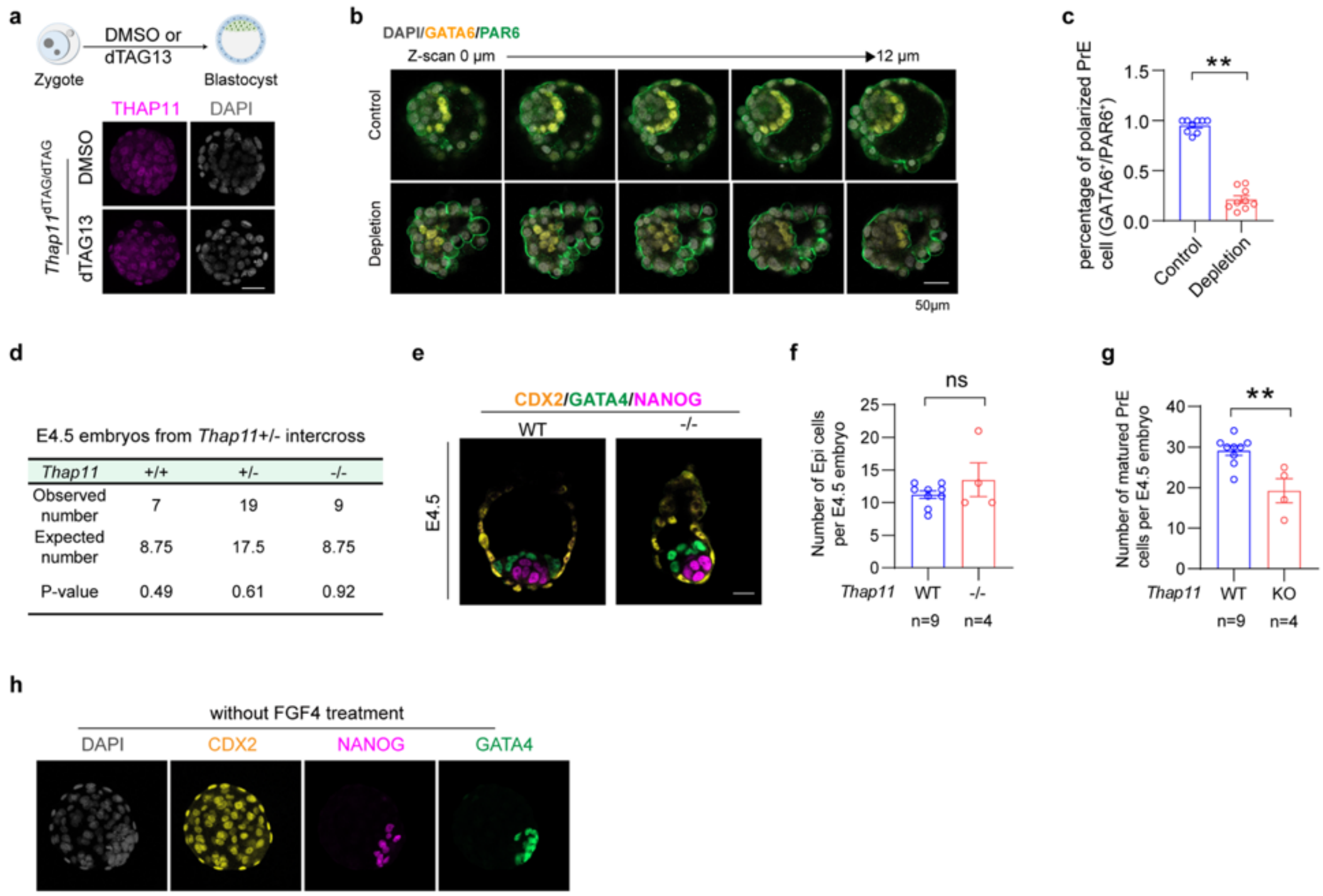


| *Thap11* | +/+ | +/- | -/- |
|---|---|---|---|
| Observed number | 7 | 19 | 9 |
| Expected number | 8.75 | 17.5 | 8.75 |
| P-value | 0.49 | 0.61 | 0.92 |

**Supplementary information, Fig. S8. THAP11 depletion causes failure of PrE polarization and maturation. a,** Immunostaining of THAP11 in *Thap11*$^{dTAG/dTAG}$ blastocysts with or without dTAG13 treatment. **b,** Individual Z-stack optical sections of GATA6 and PAR6 immunostaining in control and THAP11-depleted LB. The corresponding maximum-intensity projection is shown in Fig. 5b. **c,** Percentage of polarized PrE cells (GATA6$^{+}$/PAR6$^{+}$) per embryos of control and THAP11-depletion. Mean ± SEM; p-value, Student's t test. **, $p<0.01$. **d,** Genotype ratios of E4.5 embryos from Thap11$^{+/-}$ intercrosses. Chi-square test. **e,** Immunostaining of lineage markers CDX2, GATA4, and NANOG in WT and *Thap11*$^{-/-}$ E4.5 embryos. **f,** Number of Epi cells per E4.5 embryos of WT and *Thap11*$^{-/-}$. Mean ± SEM; p-value, Student's t test. ns, $p>0.05$. **g,** Number of matured PrE cells per E4.5 embryos of WT and *Thap11*$^{-/-}$. **h,** Immunostaining of lineage markers CDX2, NANOG, and GATA4 in WT LB embryos without FGF4 treatment. Mean ± SEM; p-value, Student's t test. **, $p<0.01$. Scale bar, 50 μm.

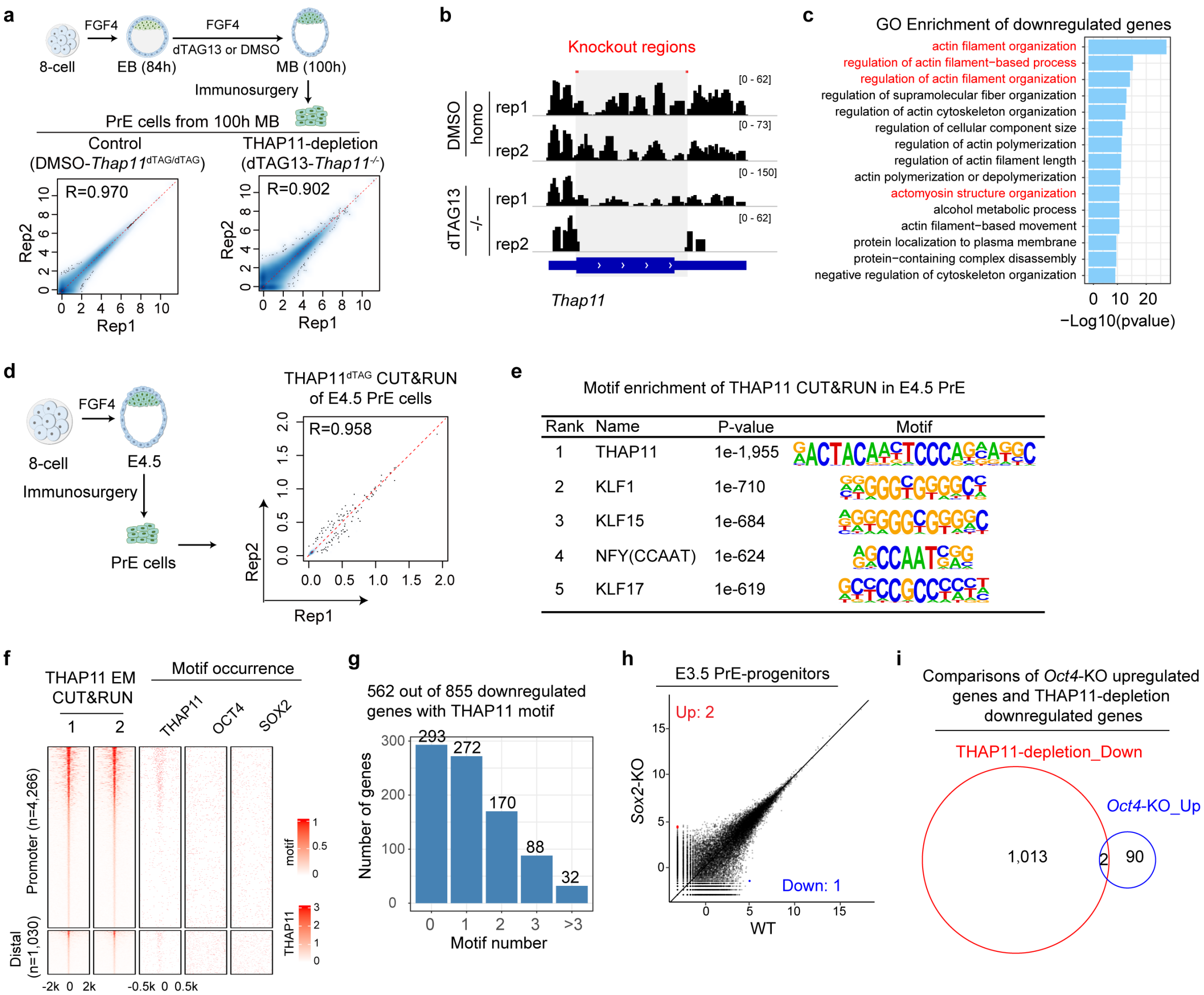


**Supplementary information, Fig. S9. THAP11 regulates polarity genes through promoter binding in PrE cells. a,** Upper panel: schematic diagram of experimental design. Lower panel: correlation of replicates for total RNA-seq in PrE cells of control (DMSO-*Thap11*$^{\mathrm{dTAG/dTAG}}$) and THAP11-depletion (dTAG13-*Thap11*$^{-/-}$). The x and y axis of the dot plots are Log (CPM+1). **b,** Genome browser view of RNA-seq at the *Thap11* genomic regions in PrE cells of control and THAP11-depletion. **c,** GO enrichment of downregulated genes (n=1,015) in response to THAP11-depletion in PrE cells. **d,** Left panel: schematic diagram of experimental design. Right panel: Correlation of replicates for THAP11 CUT&RUN in E4.5 PrE cells. Each dot represents a 5-kb bin. **e,** The top 5 TF motifs enriched in the regions bound by THAP11 in PrE cells. Note that based on the p-value, the THAP11 motif is much more highly enriched compared to the other enriched motifs. **f,** Heatmaps showing all the CUT&RUN signals and motif occurrence of THAP11 peaks at promoters and distal regions in PrE cells. **g,** THAP11 motif numbers found in the promoters of the other 855 downregulated genes without detectable CUT&RUN signal upon THAP11-depletion.

**h,** Scatter plots comparing the gene expression profiles of E3.5 PrE progenitors upon *Sox2*-KO. The x and y axis of the dot plots are $Log_2CPM$ from RNA-seq. Fold change > 2, FDR < 0.05. **i,** Venn diagram showing the overlapped genes between downregulated in THAP11-depleted PrE cells and upregulated in *Oct4*-KO PrE progenitors.

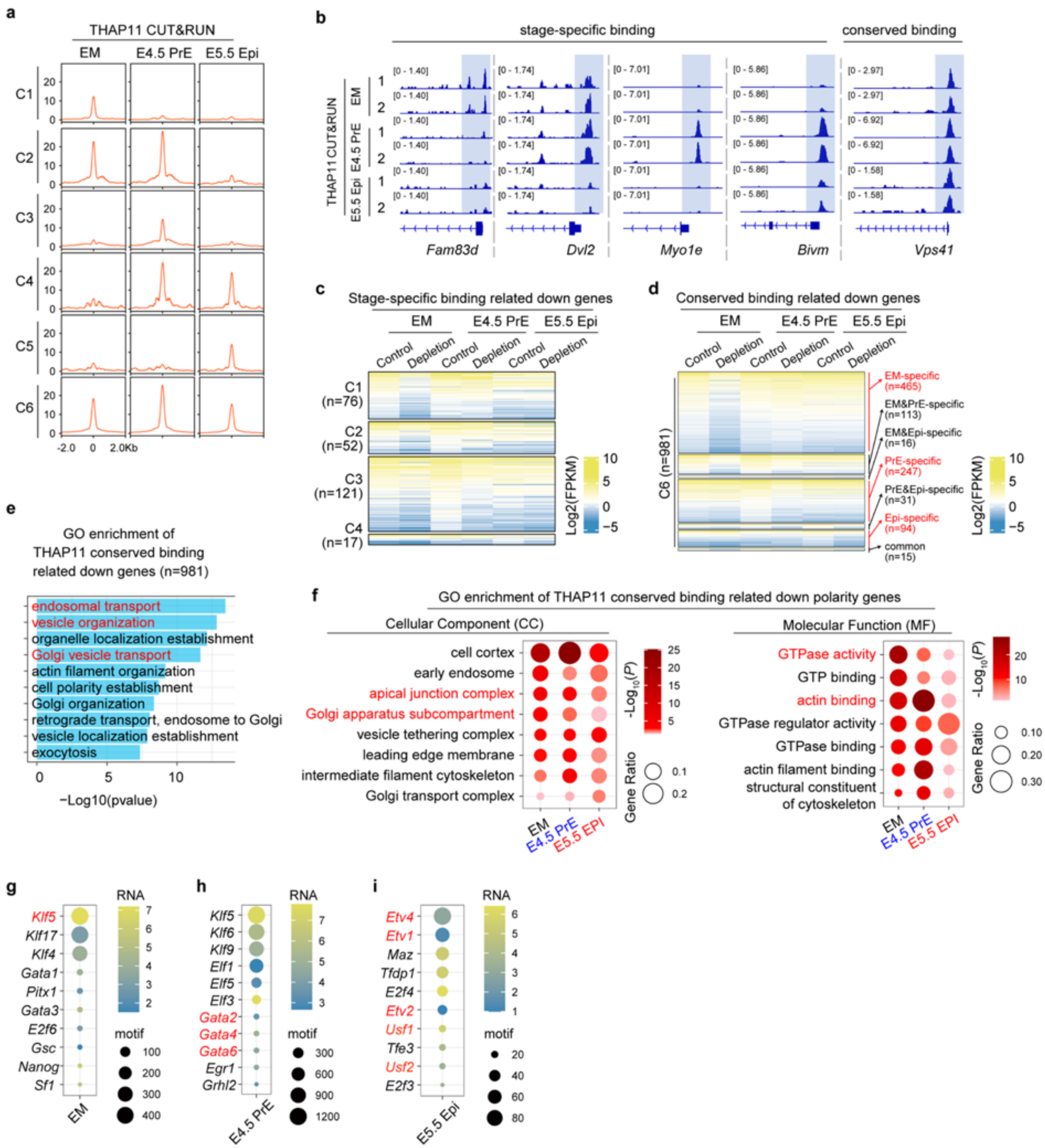


**Supplementary information, Fig. S10. Common and stage-specific THAP11 chromatin bindings regulate polarity programs. a,** Density plots of different THAP11 binding clusters (C1-C6). **b,** Genome browser view of THAP11 stage-specific and conserved targets showing CUT&RUN at the indicated stages. **c,** Heatmaps showing the gene expression of the stage-specific binding (C1-C5) associated downregulated genes (FDR<0.05, LogFC<0) in EM, E4.5 PrE, and E5.5 Epi upon THAP11 loss. **d,** Heatmaps showing the gene expression of the common binding

(C6) associated downregulated genes (FDR<0.05, LogFC<0, n=981) in EM, E4.5 PrE, and E5.5 Epi upon THAP11 loss. These genes were further sub-grouped into EM-specific (n=465), EM&PrE-specific (n=113), EM&Epi-specific (n=16), PrE-specific (n=247), PrE&Epi-specific (n=31), Epi-specific (n=94), and common (n=15) groups. **e,** Representative GO terms enriched among THAP11 conserved binding-related downregulated genes (n=981). **f,** Representative GO (CC and MF) terms enriched among THAP11 conserved binding-related downregulated polarity genes at indicated stages. **g-i,** Enrichment and expression levels of top TF motifs at the THAP11 CUT&RUN peaks of EM (g), E4.5 PrE (h), and E5.5 Epi (i).

**Supplemental tables (separated files)**

**Supplementary Table 1:** Polarity gene lists used in this study

**Supplementary Table 2:** List of differentially expressed genes in E5.5 Epi in response to THAP11 depletion

**Supplementary Table 3:** List of differentially expressed polarity genes in Epi of THAP11-depleteion and *Oct4*- or *Sox2*-KO

**Supplementary Table 4:** List of differentially expressed genes in EM of THAP11-depletion versus control

**Supplementary Table 5:** List of differentially expressed polarity genes in EM of THAP11-depleteion and *Tfap2c/Tead4*-KD

**Supplementary Table 6:** List of differentially expressed polarity genes in E8C of THAP11-overexpression

**Supplementary Table 7:** List of differentially expressed genes in PrE of THAP11-depletion versus control

**Supplementary Table 8:** List of differentially expressed genes in E3.5 PrE progenitors of *Oct4*- or *Sox2*-KO versus control

**Supplementary Table 9:** List of differentially expressed polarity genes in PrE of THAP11-depletion and *Oct4*- or *Sox2*-KO.

**Supplementary Table 10:** List of THAP11 stage-specific binding related downregulated genes.

**Supplementary Table 11:** List of THAP11 common binding related downregulated genes at indicated stages.

**Supplementary Table 12:** Candidate cofactors that may cooperate with THAP11

**Supplementary Table 13:** List of primers used in this study

**Supplementary Table 14:** ddPCR validated THAP11-dTAG knock-in copy number

**Supplementary Table 15:** Summary of the sequenced libraries in this study

**Supplementary Table 16:** Summary of the public datasets used in this study

## References

1. Zhang, C., Wang, M., Li, Y. & Zhang, Y. Profiling and functional characterization of maternal mRNA translation during mouse maternal-to-zygotic transition. *Sci. Adv.* **8**, (2022).

2. Li, H. *et al.* A complete model of mouse embryogenesis through organogenesis enabled by chemically induced embryo founder cells. *Cell* **188**, 5912-5930.e20 (2025).
